# Supplementary material for: Signal-induced enhancer activation requires Ku70 to read topoisomerase1–DNA covalent complexes
Source: Nat Struct Mol Biol. 2023 Feb 6;30(2):148–58. doi: 10.1038/s41594-022-00883-8 (PMC9935399; doi:10.1038/s41594-022-00883-8)
Supplement: Supplementary file 1 — Reporting Summary [file 41594_2022_883_MOESM1_ESM.pdf]

nature portfolio

Corresponding author(s): Michael G. Rosenfeld

Last updated by author(s): 10/18/2022

Reporting Summary

Nature Portfolio wishes to improve the reproducibility of the work that we publish. This form provides structure for consistency and transparency in reporting. For further information on Nature Portfolio policies, see our Editorial Policies and the Editorial Policy Checklist.

Statistics

For all statistical analyses, confirm that the following items are present in the figure legend, table legend, main text, or Methods section.

n/a	Confirmed	
☐	☒	The exact sample size (*n*) for each experimental group/condition, given as a discrete number and unit of measurement
☐	☒	A statement on whether measurements were taken from distinct samples or whether the same sample was measured repeatedly
☐	☒	The statistical test(s) used AND whether they are one- or two-sided<br>*Only common tests should be described solely by name; describe more complex techniques in the Methods section.*
☒	☐	A description of all covariates tested
☐	☒	A description of any assumptions or corrections, such as tests of normality and adjustment for multiple comparisons
☐	☒	A full description of the statistical parameters including central tendency (e.g. means) or other basic estimates (e.g. regression coefficient) AND variation (e.g. standard deviation) or associated estimates of uncertainty (e.g. confidence intervals)
☐	☒	For null hypothesis testing, the test statistic (e.g. *F*, *t*, *r*) with confidence intervals, effect sizes, degrees of freedom and *P* value noted<br>*Give P values as exact values whenever suitable.*
☒	☐	For Bayesian analysis, information on the choice of priors and Markov chain Monte Carlo settings
☒	☐	For hierarchical and complex designs, identification of the appropriate level for tests and full reporting of outcomes
☐	☒	Estimates of effect sizes (e.g. Cohen's *d*, Pearson's *r*), indicating how they were calculated

Our web collection on statistics for biologists contains articles on many of the points above.

Software and code

Policy information about availability of computer code

Data collection	For all high throughput sequencing, the extracted DNA libraries were sequenced with Illumina HiSeq 4000 or NovaSeq 6000 system according to the manufacturer's instructions. And DNA sequences generated by the Illumina Pipeline were aligned to the human genome (hg38) or mouse genome (mm10) assembly using Bowtie2. The data were visualized by preparing custom tracks on the University of California, Santa Cruz (UCSC) genome browser using HOMER software package.<br>Clustering plots for normalized tag densities at each genomic region were generated using HOMER and then clustered using Gene Cluster 3.0 and visualized using Java TreeView.<br>EdgeR was used to compute the significance of the differential gene expression.<br>Sequence logos were generated using WebLOGO. Gene ontology analysis was performed with Metascape.<br>The overlaps between sites identified in ChIP-seq for DNA-binding proteins and TOP1cc signals were calculated using BEDTools.
Data analysis	Analytical programs used in this paper are provided in the Methods section

For manuscripts utilizing custom algorithms or software that are central to the research but not yet described in published literature, software must be made available to editors and reviewers. We strongly encourage code deposition in a community repository (e.g. GitHub). See the Nature Portfolio guidelines for submitting code & software for further information.

Data

Policy information about availability of data

All manuscripts must include a data availability statement. This statement should provide the following information, where applicable:

- Accession codes, unique identifiers, or web links for publicly available datasets
- A description of any restrictions on data availability
- For clinical datasets or third party data, please ensure that the statement adheres to our policy

We used some published ChIP-seq data from the Gene Expression Omnibus database for DNA-PKcs under accession number GSE60270, and GRO-seq data for MCF7 with 1hr E2 treatment under accession number GSE45822. Most data are available in the main text or the supplementary materials. Whole genome sequencing datasets have been deposited to NCBI GSE135808.

Field-specific reporting

Please select the one below that is the best fit for your research. If you are not sure, read the appropriate sections before making your selection.

☒ Life sciences ☐ Behavioural & social sciences ☐ Ecological, evolutionary & environmental sciences

For a reference copy of the document with all sections, see nature.com/documents/nr-reporting-summary-flat.pdf

Life sciences study design

All studies must disclose on these points even when the disclosure is negative.

Sample size	For each experiment, the desired effect representing a difference between the populations of samples under study will be computed by estimating the mean and the variance of the distributions from an initial set of 3 biological replicates.
Data exclusions	No data was excluded.
Replication	All genome-wide experiments were replicated at least twice; and non genome-wide experiments were replicated at least 3 times.
Randomization	Random genomic regions were selected to present the distribution of Top1cc in the genome.
Blinding	All the libraries preparation for the ChIP-seq, PRO-seq, CUT&RUN assays were performed with different people to make sure the blindness. For other qPCR and Western blots experiments, the knock-down experiments and final PCR/western blot were performed by different people to make sure the blindness. For cortical neurons, the tissue preparation, library preparation were also performed with different people.

Reporting for specific materials, systems and methods

We require information from authors about some types of materials, experimental systems and methods used in many studies. Here, indicate whether each material, system or method listed is relevant to your study. If you are not sure if a list item applies to your research, read the appropriate section before selecting a response.

Materials & experimental systems

n/a	Involved in the study	
☐	☒	Antibodies
☐	☒	Eukaryotic cell lines
☒	☐	Palaeontology and archaeology
☒	☐	Animals and other organisms
☒	☐	Human research participants
☒	☐	Clinical data
☒	☐	Dual use research of concern

Methods

n/a	Involved in the study	
☐	☒	ChIP-seq
☒	☐	Flow cytometry
☒	☐	MRI-based neuroimaging

Antibodies

Antibodies used

ERα (HC20) Santa Cruz Biotechnology sc-543 (Lot# I0514)
ANTI-FLAG M2 Affinity Gel Sigma-Aldrich A2220
RNA Pol II (N20) Santa Cruz Biotechnology sc-899 (Lot# D2315)
HA Abcam ab9110(Lot#GR3177614-4)
AP2γ(H-77) Santa Cruz Biotechnology sc-8977 (Lot#G1112)
GATA3(HG3-31) Santa Cruz Biotechnology sc-268 (Lot#J0515)
CBP diagenode C15410224 (Lot#39721)

	Rad21 Abcam ab992 (Lot# GR214359-8)<br>H3K27Ac Abcam ab4729 (Lot#GR288020-1)<br>Pol II diagenode C15200004 (Lot#001-11)<br>FoxA1 diagenode C15410231 (Lot#39435)<br>BRD4 diagenode C15410337 (Lot#A2710P)<br>SMC1 Bethyl Laboratories A300-055A (A302-055A-6)<br>CTCF diagenode C15410210 (Lot#A2359-0010)<br>H3K4me2 Abcam ab7766 (Lot#GR102810-4)<br>H3K9me3 Abcam ab8898(Lot#GR3217826-1)<br>PolIISer2p Abcam ab5095(Lot#GR3225147-1)<br>H3K9me2 Cell Signaling 9753S(Lot# 4)<br>MED26 Bethyl A302-371A (Lot#A302-371A-1)<br>Med26 (13641S, Cell Signaling)<br>Top1 Bethyl A302-589A (Lot#A302-589A-1)<br>Top1cc TopoGEN TG2017-2 (Lot# 17AG15)<br>Ku70 Santa Cruz Biotechnology sc-9033 (Lot#B0416)<br>Ku-80 MyBioSource MBS8533127 (Lot#T14S11)<br>Anti-HP1g, clone 42s2 Millipore 05-690 (Lot#3224566)<br>Ku70 Bethyl A302-624A (Lot#A302-624A-1)<br>H3K4me3 Abcam Ab8580(Lot#GR3201182-1)<br>CTCF Active Motif 61311(Lot#34614003)<br>Guinea Pig anti-Rabbit IgG Antibodies-online.com ABIN101961(43047)<br>Anti-Mouse IgG Millipore 06-371(Lot#3257057)
Validation	ERα (HC20) Santa Cruz Biotechnology sc-543 (Lot# I0514) IDENTIFIER: RRID:AB_631471<br>ANTI-FLAG M2 Affinity Gel Sigma-Aldrich A2220 IDENTIFIER: RRID:AB_10063035<br>RNA Pol II (N20) Santa Cruz Biotechnology sc-899 (Lot# D2315) IDENTIFIER: RRID:AB_632359<br>HA Abcam ab9110(Lot#GR3177614-4) IDENTIFIER: RRID:AB_307019<br>AP2γ(H-77) Santa Cruz Biotechnology sc-8977 (Lot#G1112) IDENTIFIER: RRID:AB_2286995<br>GATA3(HG3-31) Santa Cruz Biotechnology sc-268 (Lot#J0515) IDENTIFIER: RRID:AB_2108591<br>CBP diagenode C15410224 (Lot#39721) IDENTIFIER: RRID:AB_2722552<br>Rad21 Abcam ab992 (Lot# GR214359-8) IDENTIFIER: RRID:AB_2314019<br>H3K27Ac Abcam ab4729 (Lot#GR288020-1) IDENTIFIER: RRID:AB_2118291<br>Pol II diagenode C15200004 (Lot#001-11) IDENTIFIER: RRID:AB_2728744<br>FoxA1 diagenode C15410231 (Lot#39435) Applications: Western Blot (WB), Immunofluorescence (IF), Immunoprecipitation (IP), ChIP/ChIP-seq, Immunohistochemistry.<br>BRD4 diagenode C15410337 (Lot#A2710P) Applications: Western Blot (WB), ELISA, ChIP/ChIP-seq.<br>SMC1 Bethyl Laboratories A300-055A (A302-055A-6) IDENTIFIER: RRID:AB_2192467<br>CTCF diagenode C15410210 (Lot#A2359-0010) IDENTIFIER: RRID:AB_2753160<br>H3K4me2 Abcam ab7766 (Lot#GR102810-4) IDENTIFIER: RRID:AB_2560996<br>H3K9me3 Abcam ab8898 (Lot#GR3217826-1) IDENTIFIER: RRID:AB_306848<br>PolIISer2p Abcam ab5095 (Lot#GR3225147-1) IDENTIFIER: RRID:AB_304749<br>H3K9me2 Cell Signaling 9753S (Lot# 4) IDENTIFIER: RRID:AB_659848<br>MED26 Bethyl A302-371A (Lot#A302-371A-1) IDENTIFIER: RRID:AB_1907254<br>MED26 Abcam Ab50619 IDENTIFIER: RRID:AB_869274<br>MED26 (13641S, Cell Signaling) DENTIFIER: RRID: AB_2798281<br>Top1 Bethyl A302-589A (Lot#A302-589A-1) IDENTIFIER: RRID:AB_2034865<br>Top1cc TopoGEN TG2017-2 (Lot# 17AG15) Applications: Western Blot (WB), ICE blot. CUT&RUN are validated in Fig.1.<br>Ku70 Santa Cruz Biotechnology sc-9033 (Lot#B0416) IDENTIFIER: RRID:AB_650476<br>Ku-80 MyBioSource MBS8533127 (Lot#T14S11) Applications: Western Blot (WB), Immunofluorescence (IF), Immunoprecipitation (IP), ChIP/ChIP-seq.<br>Anti-HP1g, clone 42s2 Millipore 05-690 (Lot#3224566) IDENTIFIER: RRID:AB_309910)<br>Ku70 Bethyl A302-624A (Lot#A302-624A-1) IDENTIFIER: RRID:AB_10554672<br>H3K4me3 Abcam qb8580 (Lot#GR3201182-1) IDENTIFIER: RRID:AB_306649<br>CTCF Active Motif 61311 (Lot#34614003) IDENTIFIER: RRID:AB_61311<br>Guinea Pig anti-Rabbit IgG Antibodies-online.com ABIN101961(43047) IDENTIFIER: RRID:AB_10775589<br>Anti-Mouse IgG Millipore 06-371(Lot#3257057) IDENTIFIER: RRID:AB_390146

Eukaryotic cell lines

Policy information about cell lines

Cell line source(s)	MCF7, 293T and LNCAP cells
Authentication	short tandem repeat (STR) was employed to determine the authentication.
Mycoplasma contamination	Mycoplasma contamination test was performed every 3 months for the all the MCF7, 293T and LNCAP cells used in our lab to make sure no contamination.
Commonly misidentified lines<br>(See ICLAC register)	N/A

ChIP-seq

Data deposition

☒ Confirm that both raw and final processed data have been deposited in a public database such as GEO.

☒ Confirm that you have deposited or provided access to graph files (e.g. BED files) for the called peaks.

Data access links
May remain private before publication.

https://www.ncbi.nlm.nih.gov/geo/query/acc.cgi?acc=GSE135808

Files in database submission

AP2y_E2.fastq.gz
AP2y_Veh.fastq.gz
BRD4_E2_exp1.fastq.gz
BRD4_E2_exp2.fastq.gz
BRD4_Veh_exp1.fastq.gz
BRD4_Veh_exp2.fastq.gz
CBP_E2.fastq.gz
CBP_Veh.fastq.gz
CBX3_siNC_E2.fastq.gz
CBX3_siNC_Veh.fastq.gz
CBX3_siTop1_E2.fastq.gz
CTCF_E2.fastq.gz
ERa_E2.fastq.gz
ERa_Veh.fastq.gz
FoxA1_E2.fastq.gz
FoxA1_Veh.fastq.gz
Gata3_E2.fastq.gz
Gata3_Veh.fastq.gz
H3K27Ac_E2.fastq.gz
H3K27Ac_Veh.fastq.gz
H3K4me3_E2.fastq.gz
H3K4me3_Veh.fastq.gz
H3K9me3_E2.fastq.gz
H3K9me3_Veh.fastq.gz
MED1_siCbx3_E2.fastq.gz
MED1_siCbx3_Veh.fastq.gz
MED1_siNC_E2.fastq.gz
MED1_siNC_Veh.fastq.gz
MED26_siCbx3_E2.fastq.gz
MED26_siCbx3_Veh.fastq.gz
MED26_siNC_E2.fastq.gz
MED26_siNC_Veh.fastq.gz
NCAPG_E2.fastq.gz
NCAPG_Veh.fastq.gz
PolIISer2P_shMed26_1_E2.fastq.gz
PolIISer2P_shMed26_1_Veh.fastq.gz
PolIISer2P_shMed26_2_E2.fastq.gz
PolIISer2P_shMed26_2_Veh.fastq.gz
PolIISer2P_shscramble_E2.fastq.gz
PolIISer2P_shscramble_Veh.fastq.gz
PolII_shMed26_1_E2.fastq.gz
PolII_shMed26_1_Veh.fastq.gz
PolII_shMed26_2_E2.fastq.gz
PolII_shMed26_2_Veh.fastq.gz
PolII_shscramble_E2.fastq.gz
PolII_shscramble_Veh.fastq.gz
PolII_siCbx3_DHT_LNCAP.fastq.gz
PolII_siCbx3_E2_MCF7.fastq.gz
PolII_siCbx3_Veh_MCF7.fastq.gz
PolII_siNC_DHT_LNCAP.fastq.gz
PolII_siNC_E2_MCF7.fastq.gz
PolII_siNC_Veh_LNCAP.fastq.gz
PolII_siNC_Veh_MCF7.fastq.gz
Rad21_E2.fastq.gz
Rad21_Veh.fastq.gz
Smc1_E2.fastq.gz
Smc1_Veh.fastq.gz
AP2y_E2.bed
AP2y_Veh.bed
BRD4_E2_exp1.bed
BRD4_E2_exp2.bed
BRD4_Veh_exp1.bed
BRD4_Veh_exp2.bed

CBP_E2.bed
CBP_Veh.bed
CBX3_siNC_E2.bed
CBX3_siNC_Veh.bed
CBX3_siTop1_E2.bed
CBX3_siTop1_Veh.bed
CTCF_E2.bed
CTCF_Veh.bed
ERa_E2.bed
ERa_Veh.bed
FoxA1_E2.bed
FoxA1_Veh.bed
Gata3_E2.bed
Gata3_Veh.bed
H3K27Ac_E2.bed
H3K27Ac_Veh.bed
H3K4me3_E2.bed
H3K4me3_Veh.bed
H3K9me3_E2.bed
H3K9me3_Veh.bed
MED1_siCbx3_E2.bed
MED1_siCbx3_Veh.bed
MED1_siNC_E2.bed
MED1_siNC_Veh.bed
MED26_siCbx3_E2.bed
MED26_siCbx3_Veh.bed
MED26_siNC_E2.bed
MED26_siNC_Veh.bed
NCAPG_E2.bed
NCAPG_Veh.bed
PolIISer2P_shMed26_1_E2.bed
PolIISer2P_shMed26_1_Veh.bed
PolIISer2P_shMed26_2_E2.bed
PolIISer2P_shMed26_2_Veh.bed
PolIISer2P_shscramble_E2.bed
PolIISer2P_shscramble_Veh.bed
PolII_shMed26_1_E2.bed
PolII_shMed26_1_Veh.bed
PolII_shMed26_2_E2.bed
PolII_shMed26_2_Veh.bed
PolII_shscramble_E2.bed
PolII_shscramble_Veh.bed
PolII_siCbx3_DHT_LNCAP.bed
PolII_siCbx3_E2_MCF7.bed
PolII_siCbx3_Veh_MCF7.bed
PolII_siNC_DHT_LNCAP.bed
PolII_siNC_E2_MCF7.bed
PolII_siNC_Veh_LNCAP.bed
PolII_siNC_Veh_MCF7.bed
Rad21_E2.bed
Rad21_Veh.bed
Smc1_E2.bed
Smc1_Veh.bed
AP2y_E2.ucsc.bigWig
AP2y_Veh.ucsc.bigWig
BRD4_E2_exp1.ucsc.bigWig
BRD4_E2_exp2.ucsc.bigWig
BRD4_Veh_exp1.ucsc.bigWig
BRD4_Veh_exp2.ucsc.bigWig
CBP_E2.ucsc.bigWig
CBP_Veh.ucsc.bigWig
CBX3_siNC_E2.ucsc.bigWig
CBX3_siNC_Veh.ucsc.bigWig
CBX3_siTop1_E2.ucsc.bigWig
CBX3_siTop1_Veh.ucsc.bigWig
CTCF_E2.ucsc.bigWig
CTCF_Veh.ucsc.bigWig
ERa_E2.ucsc.bigWig
ERa_Veh.ucsc.bigWig
FoxA1_E2.ucsc.bigWig
FoxA1_Veh.ucsc.bigWig
Gata3_E2.ucsc.bigWig
Gata3_Veh.ucsc.bigWig
H3K27Ac_E2.ucsc.bigWig
H3K27Ac_Veh.ucsc.bigWig
H3K4me3_E2.ucsc.bigWig

H3K4me3_Veh.ucsc.bigWig
H3K9me3_E2.ucsc.bigWig
H3K9me3_Veh.ucsc.bigWig
MED1_siCbx3_E2.ucsc.bigWig
MED1_siCbx3_Veh.ucsc.bigWig
MED1_siNC_E2.ucsc.bigWig
MED1_siNC_Veh.ucsc.bigWig
MED26_siCbx3_E2.ucsc.bigWig
MED26_siCbx3_Veh.ucsc.bigWig
MED26_siNC_E2.ucsc.bigWig
MED26_siNC_Veh.ucsc.bigWig
NCAPG_E2.ucsc.bigWig
NCAPG_Veh.ucsc.bigWig
PolIISer2P_shMed26_1_E2.ucsc.bigWig
PolIISer2P_shMed26_1_Veh.ucsc.bigWig
PolIISer2P_shMed26_2_E2.ucsc.bigWig
PolIISer2P_shMed26_2_Veh.ucsc.bigWig
PolIISer2P_shscramble_E2.ucsc.bigWig
PolIISer2P_shscramble_Veh.ucsc.bigWig
PolII_shMed26_1_E2.ucsc.bigWig
PolII_shMed26_1_Veh.ucsc.bigWig
PolII_shMed26_2_E2.ucsc.bigWig
PolII_shMed26_2_Veh.ucsc.bigWig
PolII_shscramble_E2.ucsc.bigWig
PolII_shscramble_Veh.ucsc.bigWig
PolII_siCbx3_DHT_LNCAP.ucsc.bigWig
PolII_siCbx3_E2_MCF7.ucsc.bigWig
PolII_siCbx3_Veh_MCF7.ucsc.bigWig
PolII_siNC_DHT_LNCAP.ucsc.bigWig
PolII_siNC_E2_MCF7.ucsc.bigWig
PolII_siNC_Veh_LNCAP.ucsc.bigWig
PolII_siNC_Veh_MCF7.ucsc.bigWig
Rad21_E2.ucsc.bigWig
Rad21_Veh.ucsc.bigWig
Smc1_E2.ucsc.bigWig
Smc1_Veh.ucsc.bigWig
H3K27ac_KCl_0min_R1_001.fastq.gz
H3K27ac_KCl_0min_R2_001.fastq.gz
H3K27ac_KCl_15min_R1_001.fastq.gz
H3K27ac_KCl_15min_R2_001.fastq.gz
H3K27ac_KCl_30min_R1_001.fastq.gz
H3K27ac_KCl_30min_R2_001.fastq.gz
H3K27ac_KCl_60min_R1_001.fastq.gz
H3K27ac_KCl_60min_R2_001.fastq.gz
H3K27me3_KCl_0min_exp1.fastq.gz
H3K27me3_KCl_0min_exp2.fastq.gz
HP1g_KCl_0min_exp2_R1_001.fastq.gz
HP1g_KCl_0min_exp2_R2_001.fastq.gz
HP1g_KCl_0min_R1_001.fastq.gz
HP1g_KCl_0min_R2_001.fastq.gz
HP1g_KCl_15min_R1_001.fastq.gz
HP1g_KCl_15min_R2_001.fastq.gz
HP1g_KCl_30min_exp2_R1_001.fastq.gz
HP1g_KCl_30min_exp2_R2_001.fastq.gz
HP1g_KCl_30min_R1_001.fastq.gz
HP1g_KCl_30min_R2_001.fastq.gz
IgG_KCl_0min_exp1.fastq.gz
IgG_KCl_0min_exp2.fastq.gz
Ku70_KCl_0min_exp2_R1_001.fastq.gz
Ku70_KCl_0min_exp2_R2_001.fastq.gz
Ku70_KCl_0min_R1_001.fastq.gz
Ku70_KCl_0min_R2_001.fastq.gz
Ku70_KCl_15min_R1_001.fastq.gz
Ku70_KCl_15min_R2_001.fastq.gz
Ku70_KCl_30min_exp2_R1_001.fastq.gz
Ku70_KCl_30min_exp2_R2_001.fastq.gz
Ku70_KCl_30min_R1_001.fastq.gz
Ku70_KCl_30min_R2_001.fastq.gz
PolII_KCl_0min_exp2_R1_001.fastq.gz
PolII_KCl_0min_exp2_R2_001.fastq.gz
PolII_KCl_0min_R1_001.fastq.gz
PolII_KCl_0min_R2_001.fastq.gz
PolII_KCl_15min_R1_001.fastq.gz
PolII_KCl_15min_R2_001.fastq.gz
PolII_KCl_180min_exp2_R1_001.fastq.gz
PolII_KCl_180min_exp2_R2_001.fastq.gz

PolII_KCl_30min_R1_001.fastq.gz
PolII_KCl_30min_R2_001.fastq.gz
PolII_KCl_60min_R1_001.fastq.gz
PolII_KCl_60min_R2_001.fastq.gz
Topo1cc_KCl_0min_exp2_R1_001.fastq.gz
Topo1cc_KCl_0min_exp2_R2_001.fastq.gz
Topo1cc_KCl_0min_R1_001.fastq.gz
Topo1cc_KCl_0min_R2_001.fastq.gz
Topo1cc_KCl_15min_R1_001.fastq.gz
Topo1cc_KCl_15min_R2_001.fastq.gz
Topo1cc_KCl_30min_exp2_R1_001.fastq.gz
Topo1cc_KCl_30min_exp2_R2_001.fastq.gz
Topo1cc_KCl_30min_R1_001.fastq.gz
Topo1cc_KCl_30min_R2_001.fastq.gz
H3K27me3_Veh_exp3_R1_001.fastq.gz
H3K27me3_Veh_exp3_R2_001.fastq.gz
H3K27me3_Veh_LNCAP_R1_001.fastq.gz
H3K27me3_Veh_LNCAP_R2_001.fastq.gz
HP1g_DHT_60min_LNCAP_exp1_R1_001.fastq.gz
HP1g_DHT_60min_LNCAP_exp1_R2_001.fastq.gz
HP1g_DHT_60min_LNCAP_exp2_R1_001.fastq.gz
HP1g_DHT_60min_LNCAP_exp2_R2_001.fastq.gz
HP1g_E2_14h_exp4_R1_001.fastq.gz
HP1g_E2_14h_exp4_R2_001.fastq.gz
HP1g_E2_1h_exp4_R1_001.fastq.gz
HP1g_E2_1h_exp4_R2_001.fastq.gz
HP1g_E2_1h_siKu70_exp6_R1_001.fastq.gz
HP1g_E2_1h_siKu70_exp6_R2_001.fastq.gz
HP1g_E2_1h_siNC_exp6_R1_001.fastq.gz
HP1g_E2_1h_siNC_exp6_R2_001.fastq.gz
HP1g_TNFa_14h_exp4_R1_001.fastq.gz
HP1g_TNFa_14h_exp4_R2_001.fastq.gz
HP1g_TNFa_1h_exp4_R1_001.fastq.gz
HP1g_TNFa_1h_exp4_R2_001.fastq.gz
HP1g_Veh_exp4_R1_001.fastq.gz
HP1g_Veh_exp4_R2_001.fastq.gz
HP1g_Veh_LNCAP_exp1_R1_001.fastq.gz
HP1g_Veh_LNCAP_exp1_R2_001.fastq.gz
HP1g_Veh_LNCAP_exp2_R1_001.fastq.gz
HP1g_Veh_LNCAP_exp2_R2_001.fastq.gz
HP1g_Veh_siKu70_exp6_R1_001.fastq.gz
HP1g_Veh_siKu70_exp6_R2_001.fastq.gz
HP1g_Veh_siNC_exp6_R1_001.fastq.gz
HP1g_Veh_siNC_exp6_R2_001.fastq.gz
IgG_Veh_exp3_R1_001.fastq.gz
IgG_Veh_exp3_R2_001.fastq.gz
IgG_Veh_exp4_R1_001.fastq.gz
IgG_Veh_exp4_R2_001.fastq.gz
IgG_Veh_LNCAP_R1_001.fastq.gz
IgG_Veh_LNCAP_R2_001.fastq.gz
Ku70_DHT_60min_LNCAP_exp1_R1_001.fastq.gz
Ku70_DHT_60min_LNCAP_exp1_R2_001.fastq.gz
Ku70_DHT_60min_LNCAP_exp2_R1_001.fastq.gz
Ku70_DHT_60min_LNCAP_exp2_R2_001.fastq.gz
Ku70_E2_14h_exp2_R1_001.fastq.gz
Ku70_E2_14h_exp2_R2_001.fastq.gz
Ku70_TNFa_14h_exp2_R1_001.fastq.gz
Ku70_TNFa_14h_exp2_R2_001.fastq.gz
Ku70_TNFa_1h_exp2_R1_001.fastq.gz
Ku70_TNFa_1h_exp2_R2_001.fastq.gz
Ku70_Veh_exp2_R1_001.fastq.gz
Ku70_Veh_exp2_R2_001.fastq.gz
Ku70_Veh_LNCAP_exp1_R1_001.fastq.gz
Ku70_Veh_LNCAP_exp1_R2_001.fastq.gz
Ku70_Veh_LNCAP_exp2_R1_001.fastq.gz
Ku70_Veh_LNCAP_exp2_R2_001.fastq.gz
Topo1cc_DHT_60min_LNCAP_exp1_R1_001.fastq.gz
Topo1cc_DHT_60min_LNCAP_exp1_R2_001.fastq.gz
Topo1cc_DHT_60min_LNCAP_exp2_R1_001.fastq.gz
Topo1cc_DHT_60min_LNCAP_exp2_R2_001.fastq.gz
Topo1cc_E2_14h_exp4_R1_001.fastq.gz
Topo1cc_E2_14h_exp4_R2_001.fastq.gz
Topo1cc_Veh_exp4_R1_001.fastq.gz
Topo1cc_Veh_exp4_R2_001.fastq.gz
Topo1cc_Veh_LNCAP_exp1_R1_001.fastq.gz
Topo1cc_Veh_LNCAP_exp1_R2_001.fastq.gz

Topo1cc_Veh_LNCAP_exp2_R1_001.fastq.gz
Topo1cc_Veh_LNCAP_exp2_R2_001.fastq.gz
Topo1_E2_14h_exp3_R1_001.fastq.gz
Topo1_E2_14h_exp3_R2_001.fastq.gz
Topo1_E2_1h_exp3_R1_001.fastq.gz
Topo1_E2_1h_exp3_R2_001.fastq.gz
Topo1_Veh_exp3_R1_001.fastq.gz
Topo1_Veh_exp3_R2_001.fastq.gz
Gata3_E2_1h_siHP1g_exp2.fastq.gz
Gata3_E2_1h_siNC_exp2.fastq.gz
Gata3_Veh_siHP1g_exp2.fastq.gz
Gata3_Veh_siNC_exp2.fastq.gz
HP1g_E2_14h_exp2.fastq.gz
HP1g_E2_14h_exp3.fastq.gz
HP1g_E2_1h_exp2.fastq.gz
HP1g_E2_1h_exp3.fastq.gz
HP1g_E2_1h_siKu70_exp5.fastq.gz
HP1g_E2_1h_siNC_exp5.fastq.gz
HP1gS83A_E2_1h_exp1.fastq.gz
HP1gS83A_E2_1h_exp2.fastq.gz
HP1gS83D_E2_1h_exp1.fastq.gz
HP1gS83D_E2_1h_exp2.fastq.gz
HP1g_Veh_exp2.fastq.gz
HP1g_Veh_exp3.fastq.gz
HP1g_Veh_siKu70_exp5.fastq.gz
HP1g_Veh_siNC_exp5.fastq.gz
Ku70_E2_1h_exp1.fastq.gz
Ku70_E2_1h_exp3.fastq.gz
Ku70_Veh_exp1.fastq.gz
Ku70_Veh_exp3.fastq.gz
Ku80_E2_1h_exp3.fastq.gz
Ku80_Veh_exp3.fastq.gz
Topo1cc_E2_1h_exp1.fastq.gz
Topo1cc_E2_1h_exp2.fastq.gz
Topo1cc_Veh_exp1.fastq.gz
Topo1cc_Veh_exp2.fastq.gz
H3K27ac_KCl_0min.ucsc.bedGraph.gz
H3K27ac_KCl_15min.ucsc.bedGraph.gz
H3K27ac_KCl_30min.ucsc.bedGraph.gz
H3K27ac_KCl_60min.ucsc.bedGraph.gz
H3K27me3_KCl_0min_exp1.ucsc.bedGraph.gz
H3K27me3_KCl_0min_exp2.ucsc.bedGraph.gz
HP1g_KCl_0min_exp2.ucsc.bedGraph.gz
HP1g_KCl_0min.ucsc.bedGraph.gz
HP1g_KCl_15min.ucsc.bedGraph.gz
HP1g_KCl_30min_exp2.ucsc.bedGraph.gz
HP1g_KCl_30min.ucsc.bedGraph.gz
IgG_KCl_0min_exp1.ucsc.bedGraph.gz
IgG_KCl_0min_exp2.ucsc.bedGraph.gz
Ku70_KCl_0min_exp2.ucsc.bedGraph.gz
Ku70_KCl_0min.ucsc.bedGraph.gz
Ku70_KCl_15min.ucsc.bedGraph.gz
Ku70_KCl_30min_exp2.ucsc.bedGraph.gz
Ku70_KCl_30min.ucsc.bedGraph.gz
PolII_KCl_0min_exp2.ucsc.bedGraph.gz
PolII_KCl_0min.ucsc.bedGraph.gz
PolII_KCl_15min.ucsc.bedGraph.gz
PolII_KCl_180min_exp2.ucsc.bedGraph.gz
PolII_KCl_30min.ucsc.bedGraph.gz
PolII_KCl_60min.ucsc.bedGraph.gz
Topo1cc_KCl_0min_exp2.ucsc.bedGraph.gz
Topo1cc_KCl_0min.ucsc.bedGraph.gz
Topo1cc_KCl_15min.ucsc.bedGraph.gz
Topo1cc_KCl_30min_exp2.ucsc.bedGraph.gz
Topo1cc_KCl_30min.ucsc.bedGraph.gz
H3K27ac_KCl_0min.bed
H3K27ac_KCl_15min.bed
H3K27ac_KCl_30min.bed
H3K27ac_KCl_60min.bed
H3K27me3_KCl_0min_exp1.bed
H3K27me3_KCl_0min_exp2.bed
HP1g_KCl_0min.bed
HP1g_KCl_0min_exp2.bed
HP1g_KCl_15min.bed
HP1g_KCl_30min.bed
HP1g_KCl_30min_exp2.bed

IgG_KCl_0min_exp1.bed
IgG_KCl_0min_exp2.bed
Ku70_KCl_0min.bed
Ku70_KCl_0min_exp2.bed
Ku70_KCl_15min.bed
Ku70_KCl_30min.bed
Ku70_KCl_30min_exp2.bed
PolII_KCl_0min.bed
PolII_KCl_0min_exp2.bed
PolII_KCl_15min.bed
PolII_KCl_180min_exp2.bed
PolII_KCl_30min.bed
PolII_KCl_60min.bed
Topo1cc_KCl_0min.bed
Topo1cc_KCl_0min_exp2.bed
Topo1cc_KCl_15min.bed
Topo1cc_KCl_30min.bed
Topo1cc_KCl_30min_exp2.bed
Gata3_E2_1h_siHP1g_exp2.bed
Gata3_E2_1h_siHp1g_exp2.fastq.gz
Gata3_E2_1h_siHp1g_exp2.ucsc.bedGraph.gz
Gata3_E2_1h_siNC_exp2.bed
Gata3_E2_1h_siNC_exp2.fastq.gz
Gata3_E2_1h_siNC_exp2.ucsc.bedGraph.gz
Gata3_Veh_siHP1g_exp2.bed
Gata3_Veh_siHP1g_exp2.fastq.gz
Gata3_Veh_siHP1g_exp2.ucsc.bedGraph.gz
Gata3_Veh_siNC_exp2.bed
Gata3_Veh_siNC_exp2.fastq.gz
Gata3_Veh_siNC_exp2.ucsc.bedGraph.gz
HP1g_E2_14h_exp2.bed
HP1g_E2_14h_exp2.fastq.gz
HP1g_E2_14h_exp2.ucsc.bedGraph.gz
HP1g_E2_14h_exp3.bed
HP1g_E2_14h_exp3.fastq.gz
HP1g_E2_14h_exp3.ucsc.bedGraph.gz
HP1g_E2_1h_exp2.bed
HP1g_E2_1h_exp2.fastq.gz
HP1g_E2_1h_exp2.ucsc.bedGraph.gz
HP1g_E2_1h_exp3.bed
HP1g_E2_1h_exp3.fastq.gz
HP1g_E2_1h_exp3.ucsc.bedGraph.gz
HP1g_E2_1h_siKu70_exp5.bed
HP1g_E2_1h_siKu70_exp5.fastq.gz
HP1g_E2_1h_siKu70_exp5.ucsc.bedGraph.gz
HP1g_E2_1h_siNC_exp5.bed
HP1g_E2_1h_siNC_exp5.fastq.gz
HP1g_E2_1h_siNC_exp5.ucsc.bedGraph.gz
HP1gS83A_E2_1h_exp1.bed
HP1gS83A_E2_1h_exp1.fastq.gz
HP1gS83A_E2_1h_exp1.ucsc.bedGraph.gz
HP1gS83A_E2_1h_exp2.bed
HP1gS83A_E2_1h_exp2.fastq.gz
HP1gS83A_E2_1h_exp2.ucsc.bedGraph.gz
HP1gS83D_E2_1h_exp1.bed
HP1gS83D_E2_1h_exp1.fastq.gz
HP1gS83D_E2_1h_exp1.ucsc.bedGraph.gz
HP1gS83D_E2_1h_exp2.bed
HP1gS83D_E2_1h_exp2.fastq.gz
HP1gS83D_E2_1h_exp2.ucsc.bedGraph.gz
HP1g_Veh_exp2.bed
HP1g_Veh_exp2.fastq.gz
HP1g_Veh_exp2.ucsc.bedGraph.gz
HP1g_Veh_exp3.bed
HP1g_Veh_exp3.fastq.gz
HP1g_Veh_exp3.ucsc.bedGraph.gz
HP1g_Veh_siKu70_exp5.bed
HP1g_Veh_siKu70_exp5.fastq.gz
HP1g_Veh_siKu70_exp5.ucsc.bedGraph.gz
HP1g_Veh_siNC_exp5.bed
HP1g_Veh_siNC_exp5.fastq.gz
HP1g_Veh_siNC_exp5.ucsc.bedGraph.gz
Ku70_E2_1h_exp1.bed
Ku70_E2_1h_exp1.fastq.gz
Ku70_E2_1h_exp1.ucsc.bedGraph.gz
Ku70_E2_1h_exp3.bed

Ku70_E2_1h_exp3.fastq.gz
Ku70_E2_1h_exp3.ucsc.bedGraph.gz
Ku70_Veh_exp1.bed
Ku70_Veh_exp1.fastq.gz
Ku70_Veh_exp1.ucsc.bedGraph.gz
Ku70_Veh_exp3.bed
Ku70_Veh_exp3.fastq.gz
Ku70_Veh_exp3.ucsc.bedGraph.gz
Ku80_E2_1h_exp3.bed
Ku80_E2_1h_exp3.fastq.gz
Ku80_E2_1h_exp3.ucsc.bedGraph.gz
Ku80_Veh_exp3.bed
Ku80_Veh_exp3.fastq.gz
Ku80_Veh_exp3.ucsc.bedGraph.gz
Topo1cc_E2_1h_exp1.bed
Topo1cc_E2_1h_exp1.fastq.gz
Topo1cc_E2_1h_exp1.ucsc.bedGraph.gz
Topo1cc_E2_1h_exp2.bed
Topo1cc_E2_1h_exp2.fastq.gz
Topo1cc_E2_1h_exp2.ucsc.bedGraph.gz
Topo1cc_Veh_exp1.bed
Topo1cc_Veh_exp1.fastq.gz
Topo1cc_Veh_exp1.ucsc.bedGraph.gz
Topo1cc_Veh_exp2.bed
Topo1cc_Veh_exp2.fastq.gz
Topo1cc_Veh_exp2.ucsc.bedGraph.gz
H3K27me3_Veh_exp3.bed
H3K27me3_Veh_exp3_R1_001.fastq.gz
H3K27me3_Veh_exp3_R2_001.fastq.gz
H3K27me3_Veh_exp3.ucsc.bedGraph.gz
H3K27me3_Veh_LNCAP.bed
H3K27me3_Veh_LNCAP_R1_001.fastq.gz
H3K27me3_Veh_LNCAP_R2_001.fastq.gz
H3K27me3_Veh_LNCAP.ucsc.bedGraph.gz
HP1g_DHT_60min_LNCAP_exp1.bed
HP1g_DHT_60min_LNCAP_exp1_R1_001.fastq.gz
HP1g_DHT_60min_LNCAP_exp1_R2_001.fastq.gz
HP1g_DHT_60min_LNCAP_exp1.ucsc.bedGraph.gz
HP1g_DHT_60min_LNCAP_exp2.bed
HP1g_DHT_60min_LNCAP_exp2_R1_001.fastq.gz
HP1g_DHT_60min_LNCAP_exp2_R2_001.fastq.gz
HP1g_DHT_60min_LNCAP_exp2.ucsc.bedGraph.gz
HP1g_E2_14h_exp4.bed
HP1g_E2_14h_exp4_R1_001.fastq.gz
HP1g_E2_14h_exp4_R2_001.fastq.gz
HP1g_E2_14h_exp4.ucsc.bedGraph.gz
HP1g_E2_1h_exp4.bed
HP1g_E2_1h_exp4_R1_001.fastq.gz
HP1g_E2_1h_exp4_R2_001.fastq.gz
HP1g_E2_1h_exp4.ucsc.bedGraph.gz
HP1g_E2_1h_siKu70_exp6.bed
HP1g_E2_1h_siKu70_exp6_R1_001.fastq.gz
HP1g_E2_1h_siKu70_exp6_R2_001.fastq.gz
HP1g_E2_1h_siKu70_exp6.ucsc.bedGraph.gz
HP1g_E2_1h_siNC_exp6.bed
HP1g_E2_1h_siNC_exp6_R1_001.fastq.gz
HP1g_E2_1h_siNC_exp6_R2_001.fastq.gz
HP1g_E2_1h_siNC_exp6.ucsc.bedGraph.gz
HP1g_TNFa_14h_exp4.bed
HP1g_TNFa_14h_exp4_R1_001.fastq.gz
HP1g_TNFa_14h_exp4_R2_001.fastq.gz
HP1g_TNFa_1h_exp4.bed
HP1g_TNFa_1h_exp4_R1_001.fastq.gz
HP1g_TNFa_1h_exp4_R2_001.fastq.gz
HP1g_TNFa_1h_exp4.ucsc.bedGraph.gz
HP1g_Veh_exp4.bed
HP1g_Veh_exp4_R1_001.fastq.gz
HP1g_Veh_exp4_R2_001.fastq.gz
HP1g_Veh_exp4.ucsc.bedGraph.gz
HP1g_Veh_LNCAP_exp1.bed
HP1g_Veh_LNCAP_exp1_R1_001.fastq.gz
HP1g_Veh_LNCAP_exp1_R2_001.fastq.gz
HP1g_Veh_LNCAP_exp1.ucsc.bedGraph.gz
HP1g_Veh_LNCAP_exp2.bed
HP1g_Veh_LNCAP_exp2_R1_001.fastq.gz
HP1g_Veh_LNCAP_exp2_R2_001.fastq.gz

HP1g_Veh_LNCAP_exp2.ucsc.bedGraph.gz
HP1g_Veh_siKu70_exp6.bed
HP1g_Veh_siKu70_exp6_R1_001.fastq.gz
HP1g_Veh_siKu70_exp6_R2_001.fastq.gz
HP1g_Veh_siKu70_exp6.ucsc.bedGraph.gz
HP1g_Veh_siNC_exp6.bed
HP1g_Veh_siNC_exp6_R1_001.fastq.gz
HP1g_Veh_siNC_exp6_R2_001.fastq.gz
HP1g_Veh_siNC_exp6.ucsc.bedGraph.gz
IgG_Veh_exp4.bed
IgG_Veh_exp4_R1_001.fastq.gz
IgG_Veh_exp4_R2_001.fastq.gz
IgG_Veh_exp4.ucsc.bedGraph.gz
IgG_Veh_LNCAP.bed
IgG_Veh_LNCAP_R1_001.fastq.gz
IgG_Veh_LNCAP_R2_001.fastq.gz
IgG_Veh_LNCAP.ucsc.bedGraph.gz
Ku70_DHT_60min_LNCAP_exp1.bed
Ku70_DHT_60min_LNCAP_exp1_R1_001.fastq.gz
Ku70_DHT_60min_LNCAP_exp1_R2_001.fastq.gz
Ku70_DHT_60min_LNCAP_exp1.ucsc.bedGraph.gz
Ku70_DHT_60min_LNCAP_exp2.bed
Ku70_DHT_60min_LNCAP_exp2_R1_001.fastq.gz
Ku70_DHT_60min_LNCAP_exp2_R2_001.fastq.gz
Ku70_DHT_60min_LNCAP_exp2.ucsc.bedGraph.gz
Ku70_E2_14h_exp2.bed
Ku70_E2_14h_exp2_R1_001.fastq.gz
Ku70_E2_14h_exp2_R2_001.fastq.gz
Ku70_E2_14h_exp2.ucsc.bedGraph.gz
Ku70_TNFa_14h_exp2.bed
Ku70_TNFa_14h_exp2_R1_001.fastq.gz
Ku70_TNFa_14h_exp2_R2_001.fastq.gz
Ku70_TNFa_14h_exp2.ucsc.bedGraph.gz
Ku70_TNFa_1h_exp2.bed
Ku70_TNFa_1h_exp2_R1_001.fastq.gz
Ku70_TNFa_1h_exp2_R2_001.fastq.gz
Ku70_TNFa_1h_exp2.ucsc.bedGraph.gz
Ku70_Veh_exp2.bed
Ku70_Veh_exp2_R1_001.fastq.gz
Ku70_Veh_exp2_R2_001.fastq.gz
Ku70_Veh_exp2.ucsc.bedGraph.gz
Ku70_Veh_LNCAP_exp1.bed
Ku70_Veh_LNCAP_exp1_R1_001.fastq.gz
Ku70_Veh_LNCAP_exp1_R2_001.fastq.gz
Ku70_Veh_LNCAP_exp1.ucsc.bedGraph.gz
Ku70_Veh_LNCAP_exp2.bed
Ku70_Veh_LNCAP_exp2_R1_001.fastq.gz
Ku70_Veh_LNCAP_exp2_R2_001.fastq.gz
Ku70_Veh_LNCAP_exp2.ucsc.bedGraph.gz
Topo1cc_DHT_60min_LNCAP_exp1.bed
Topo1cc_DHT_60min_LNCAP_exp1_R1_001.fastq.gz
Topo1cc_DHT_60min_LNCAP_exp1_R2_001.fastq.gz
Topo1cc_DHT_60min_LNCAP_exp1.ucsc.bedGraph.gz
Topo1cc_DHT_60min_LNCAP_exp2.bed
Topo1cc_DHT_60min_LNCAP_exp2_R1_001.fastq.gz
Topo1cc_DHT_60min_LNCAP_exp2_R2_001.fastq.gz
Topo1cc_DHT_60min_LNCAP_exp2.ucsc.bedGraph.gz
Topo1cc_E2_14h_exp4.bed
Topo1cc_E2_14h_exp4_R1_001.fastq.gz
Topo1cc_E2_14h_exp4_R2_001.fastq.gz
Topo1cc_E2_14h_exp4.ucsc.bedGraph.gz
Topo1cc_Veh_exp4.bed
Topo1cc_Veh_exp4_R1_001.fastq.gz
Topo1cc_Veh_exp4_R2_001.fastq.gz
Topo1cc_Veh_exp4.ucsc.bedGraph.gz
Topo1cc_Veh_LNCAP_exp1.bed
Topo1cc_Veh_LNCAP_exp1_R1_001.fastq.gz
Topo1cc_Veh_LNCAP_exp1_R2_001.fastq.gz
Topo1cc_Veh_LNCAP_exp1.ucsc.bedGraph.gz
Topo1cc_Veh_LNCAP_exp2.bed
Topo1cc_Veh_LNCAP_exp2_R1_001.fastq.gz
Topo1cc_Veh_LNCAP_exp2_R2_001.fastq.gz
Topo1cc_Veh_LNCAP_exp2.ucsc.bedGraph.gz
Topo1_E2_14h_exp3.bed
Topo1_E2_14h_exp3_R1_001.fastq.gz
Topo1_E2_14h_exp3_R2_001.fastq.gz

Topo1_E2_14h_exp3.ucsc.bedGraph.gz
Topo1_E2_1h_exp3.bed
Topo1_E2_1h_exp3_R1_001.fastq.gz
Topo1_E2_1h_exp3_R2_001.fastq.gz
Topo1_E2_1h_exp3.ucsc.bedGraph.gz
Topo1_Veh_exp3.bed
Topo1_Veh_exp3_R1_001.fastq.gz
Topo1_Veh_exp3_R2_001.fastq.gz
Topo1_Veh_exp3.ucsc.bedGraph.gz

Genome browser session
(e.g. UCSC)

For human cancer cell data:
http://genome.ucsc.edu/s/yuliangtan/Topo1cc_hg38

For cortical neuron data:
http://genome.ucsc.edu/s/yuliangtan/Topo1cc_mm10

Methodology

Replicates

All experiments were replicated at least twice.

Sequencing depth

At least 30, 000,000 reads were detected at most of the sequencing data.

Antibodies

ERα (HC20) Santa Cruz Biotechnology sc-543 (Lot# I0514)
RNA Pol II (N20) Santa Cruz Biotechnology sc-899 (Lot# D2315)
AP2γ(H-77) Santa Cruz Biotechnology sc-8977 (Lot#G1112)
GATA3(HG3-31) Santa Cruz Biotechnology sc-268 (Lot#J0515)
CBP diagenode C15410224 (Lot#39721)
Rad21 Abcam ab992 (Lot# GR214359-8)
H3K27Ac Abcam ab4729 (Lot#GR288020-1)
Pol II diagenode C15200004 (Lot#001-11)
FoxA1 diagenode C15410231 (Lot#39435)
BRD4 diagenode C15410337 (Lot#A2710P)
SMC1 Bethyl Laboratories A300-055A (A302-055A-6)
H3K4me2 Abcam ab7766 (Lot#GR102810-4)
H3K9me3 Abcam ab8898(Lot#GR3217826-1)
PolIISer2p Abcam ab5095(Lot#GR3225147-1)
H3K9me2 Cell Signaling 9753S(Lot# 4)
MED26 (13641S, Cell Signaling)
TOP1 Bethyl A302-589A (Lot#A302-589A-1)
TOP1cc TopoGEN TG2017-2 (Lot# 17AG15)
Ku-80 MyBioSource MBS8533127 (Lot#T14S11)
Anti-HP1g, clone 42s2 Millipore 05-690 (Lot#3224566)
Ku70 Bethyl A302-624A (Lot#A302-624A-1)
H3K4me3 Abcam Ab8580(Lot#GR3201182-1)
CTCF Active Motif 61311(Lot#34614003)

Peak calling parameters

For TFs: findPeaks -style factor -o auto
For histones: findPeaks -style histone -o auto

Data quality

maximum tags considered per bp = 1.0
effective number of tags used for normalization = 10000000.0
Peaks have been centered at maximum tag pile-up
FDR rate threshold = 0.001000000
FDR tag threshold = 20.0
size of region used for local filtering = 10000
Fold over local region required = 4.00
Poisson p-value over local region required = 1.00e-04
Maximum fold under expected unique positions for tags = 2.00
Putative peaks filtered for being too clonal = 0

Software

DNA sequences generated by the Illumina Pipeline were aligned to the human genome (hg38) or mouse genome (mm10) assembly using Bowtie2. The data were visualized by preparing custom tracks on the University of California, Santa Cruz (UCSC) genome browser using HOMER software package.
Clustering plots for normalized tag densities at each genomic region were generated using HOMER and then clustered using Gene Cluster 3.0 and visualized using Java TreeView.
Sequence logos were generated using WebLOGO. Gene ontology analysis was performed with Metascape.
The overlaps between sites identified in ChIP-seq for DNA-binding proteins and TOP1cc signals were calculated using BEDTools.
