# Supplementary material for: Signal-induced enhancer activation requires Ku70 to read topoisomerase1–DNA covalent complexes
Source: Nat Struct Mol Biol. 2023 Feb 6;30(2):148–58. doi: 10.1038/s41594-022-00883-8 (PMC9935399; doi:10.1038/s41594-022-00883-8)
Supplement: Source Data Fig. 1b,d — Source data for Fig. 1b,d. [file 41594_2022_883_MOESM4_ESM.pdf]

Fig.1b

	ATAC	SMC1	RAD21	CTCF	H3K4me3	H3K9me3	H3K27Ac	H3K4me2	H3K27me3	H2Az	H4K16Ac	H4K20me1
Numbers of TOP1cc Peaks	12451	10922	9036	4694	6331	896	15103	14488	253	9995	13326	1097

Fig.1d

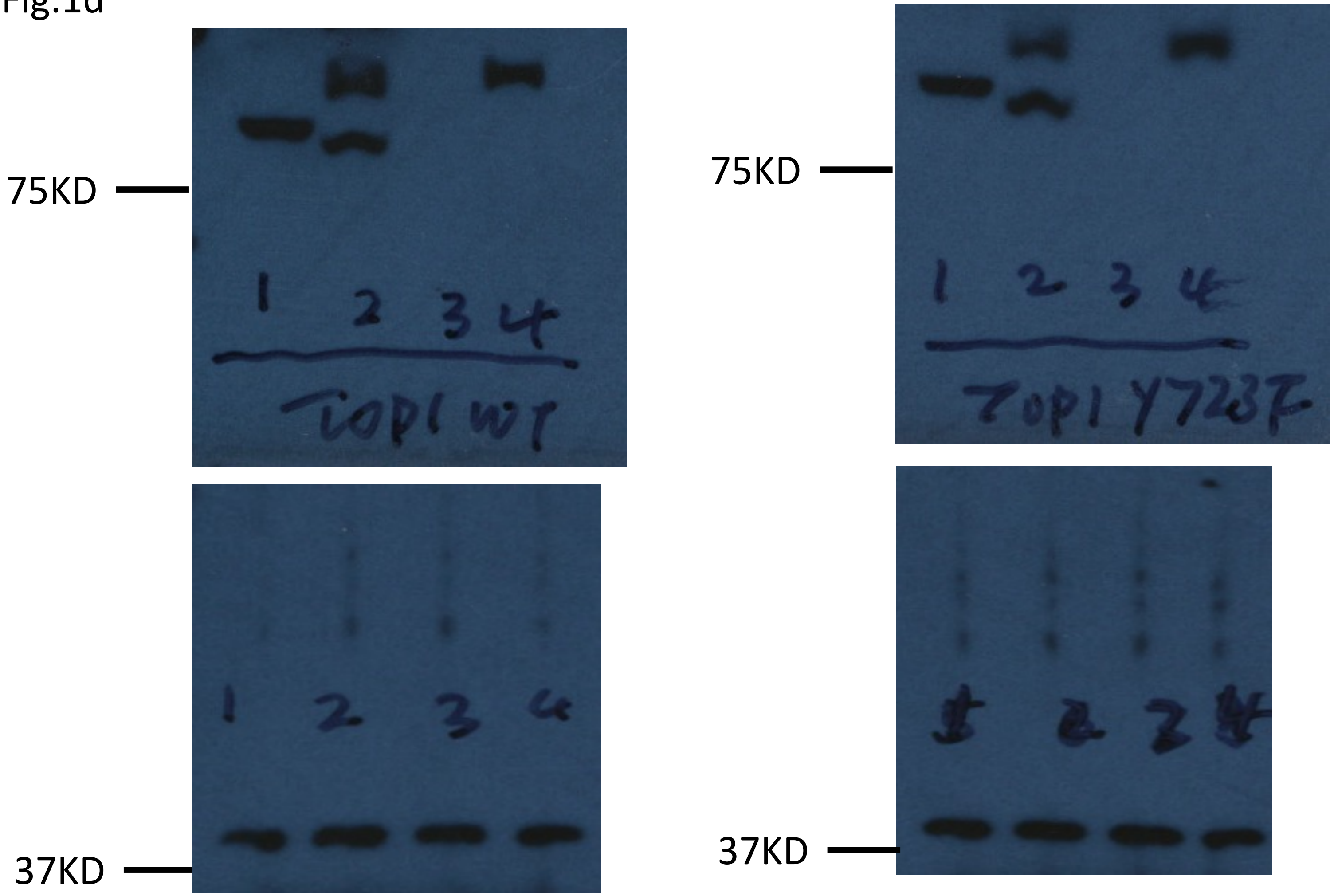

1: CTRL gRNA DOX -
2: CTRL gRNA DOX +
3: Top1 gRNA DOX -
4: Top1 gRNA DOX +
