# Supplementary material for: Signal-induced enhancer activation requires Ku70 to read topoisomerase1–DNA covalent complexes
Source: Nat Struct Mol Biol. 2023 Feb 6;30(2):148–58. doi: 10.1038/s41594-022-00883-8 (PMC9935399; doi:10.1038/s41594-022-00883-8)
Supplement: Source Data Fig. 2c,e–f — Source data for Fig. 2c,e–f. [file 41594_2022_883_MOESM5_ESM.pdf]

Fig.2c

Tff1 enhancer RNA	Ctrl gRNA			Top1 gRNA			Ctrl gRNA+TOP1wt			Top1p gRNA+TOP1wt		
Veh	30.7434	30.3447	30.0745	30.0988	30.1051	29.9699	31.5589	31.7392	31.3467	30.4551	30.0598	29.581
E2	27.7381	27.4425	27.257	30.3438	30.5551	29.8627	28.2824	28.8944	28.7488	28.3487	28.2801	28.6079
Tff1 enhancer RNA	Ctrl gRNA			Top1 gRNA			Ctrl gRNA+TOP1Y723F			Top1 gRNA+TOP1Y723F		
Veh	31.0588	30.4115	31.1347	31.2679	30.2473	30.6532	30.3148	30.4984	29.4187	29.8362	29.792	29.7208
E2	27.6647	27.6716	27.9447	29.8459	29.9313	30.5178	28.9493	28.4241	28.1241	29.5582	30.0564	29.5668
Greb1 enhancer RNA	Ctrl gRNA			Top1 gRNA			Ctrl gRNA+TOP1wt			Top1 gRNA+TOP1wt		
Veh	29.5415	29.107	28.8297	30.2717	30.1076	29.8103	30.896	30.3272	30.3265	28.9953	29.3456	29.2663
E2	27.2226	27.249	27.1241	29.8072	30.8419	30.2547	27.8924	27.9434	28.3649	26.4636	26.415	26.0592
Greb1 enhancer RNA	Ctrl gRNA			Top1 gRNA			Ctrl gRNA+TOP1Y723F			Top1 gRNA+TOP1Y723F		
Veh	32.1317	31.4529	31.8146	31.5731	31.3059	31.6099	31.3371	31.877	30.4073	31.2756	30.9844	30.9309
E2	28.0093	27.4344	27.957	31.0692	30.9817	31.6685	28.8717	28.8548	28.2234	29.9076	29.8539	30.1048
GAPDH WT	Ctrl gRNA			Top1 gRNA			Ctrl gRNA+TOP1wt			Top1p gRNA+TOP1wt		
Veh	19.8342	19.9367	19.8082	20.184	20.008	20.1231	21.3772	21.0674	21.3414	20.0013	19.7739	19.468
E2	20.1821	20.2778	20.2863	20.6566	20.5498	20.0617	20.9756	21.165	21.6533	19.3248	19.5798	19.3479
GAPDH Y723F	Ctrl gRNA			Top1 gRNA			Ctrl gRNA+TOP1wt			Top1p gRNA+TOP1wt		
Veh	21.2214	21.0442	21.5489	21.6588	21.2092	21.7635	21.1557	21.2056	20.4022	20.8221	20.6991	20.8183
E2	20.4533	20.2698	20.9864	21.3825	20.9766	21.8679	21.5649	21.1253	21.128	20.884	21.1534	20.845

Fig.2e

FoxC1	siNC			siTop1		
Veh	29.6849	29.7959	28.8874	29.8454	29.5674	30.263
E2	27.8247	28.3695	27.7598	29.2396	29.1515	29.6721
Greb1	siNC			siTop1		
Veh	25.3366	25.9371	25.9712	25.6995	25.9119	26.1364
Acute E2	23.8902	23.9549	23.868	25.5626	25.5441	25.583
KCNK5	siNC			siTop1		
Veh	28.9357	29.4611	29.4995	29.1796	29.2502	29.4112
Acute E2	25.1022	25.1451	25.1599	26.9462	27.7516	27.2649
Nrip1	siNC			siTop1		
Veh	27.4841	27.9175	27.8048	27.6543	27.7772	27.6944
Acute E2	26.5885	26.5946	26.5901	28.2988	28.0861	28.5658
P2ry2	siNC			siTop1		
Veh	25.943	25.9357	25.8597	25.9353	26.0011	25.983
Acute E2	24.6647	24.879	24.5725	26.4657	26.076	26.654
Pgr	siNC			siTop1		
Veh	29.944	29.6732	29.9601	29.5249	29.3555	29.7054
Acute E2	25.2472	25.6536	25.6112	27.4189	27.2161	27.785
GAPDH	siNC			siTop1		
Veh	18.3460	18.4692	18.5924	19.2389	18.8266	18.4143
Acute E2	19.5752	19.8070	20.0388	19.6882	19.4900	19.2918

Fig.2f

FoxC1	siNC			siTop1		
Veh	30.8060	30.3624	30.5947	30.9946	31.1628	31.0404
E2	28.8686	28.9420	28.0666	28.7520	28.3115	28.9928
Greb1	siNC			siTop1		
Veh	26.0103	26.5312	26.5033	27.1830	27.2081	27.4061
E2	22.8415	22.1470	22.3874	22.5283	22.4862	22.7350
KCNK5	siNC			siTop1		
Veh	27.9587	28.0875	28.0385	29.4933	29.1043	29.4735
E2	24.5030	25.1303	24.7989	25.0030	25.7384	25.4085
Nrip1	siNC			siTop1		
Veh	27.8245	27.8697	27.8074	27.9856	27.9506	28.0907
E2	25.7532	26.1701	25.6988	25.7234	25.6770	26.0465
P2ry2	siNC			siTop1		
Veh	26.0559	25.8524	25.6825	27.0265	27.0454	27.3733
E2	26.2927	26.9815	26.9538	26.3202	26.0201	26.6523
Pgr	siNC			siTop1		
Veh	24.3637	24.0588	24.3076	24.9678	26.8643	25.6161
E2	24.1719	24.5601	24.3070	24.3382	24.1591	24.6665
GAPDH	siNC			siTop1		
Veh	19.5656	19.3852	19.4093	20.0169	19.9417	20.0111
E2	19.6378	19.9172	19.5799	19.6193	19.5723	19.8028
