# Supplementary material for: Signal-induced enhancer activation requires Ku70 to read topoisomerase1–DNA covalent complexes
Source: Nat Struct Mol Biol. 2023 Feb 6;30(2):148–58. doi: 10.1038/s41594-022-00883-8 (PMC9935399; doi:10.1038/s41594-022-00883-8)
Supplement: Source Data Fig. 3b,e–f — Unprocessed western blots for Fig. 3b,e–f. [file 41594_2022_883_MOESM6_ESM.pdf]

Fig.3b

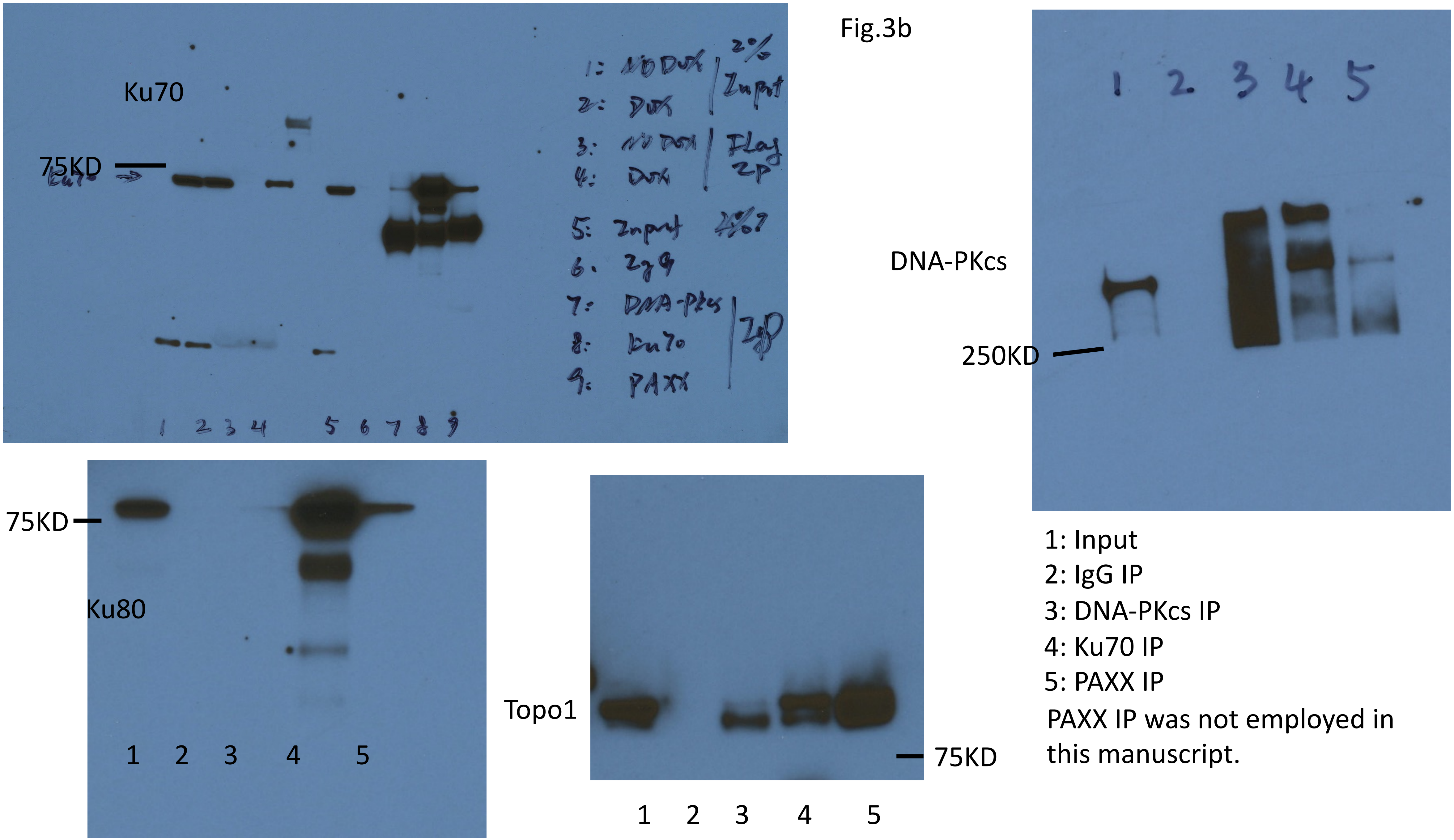

1: Input
2: IgG IP
3: DNA-PKcs IP
4: Ku70 IP
5: PAXX IP

PAXX IP was not employed in this manuscript.

Fig.3e

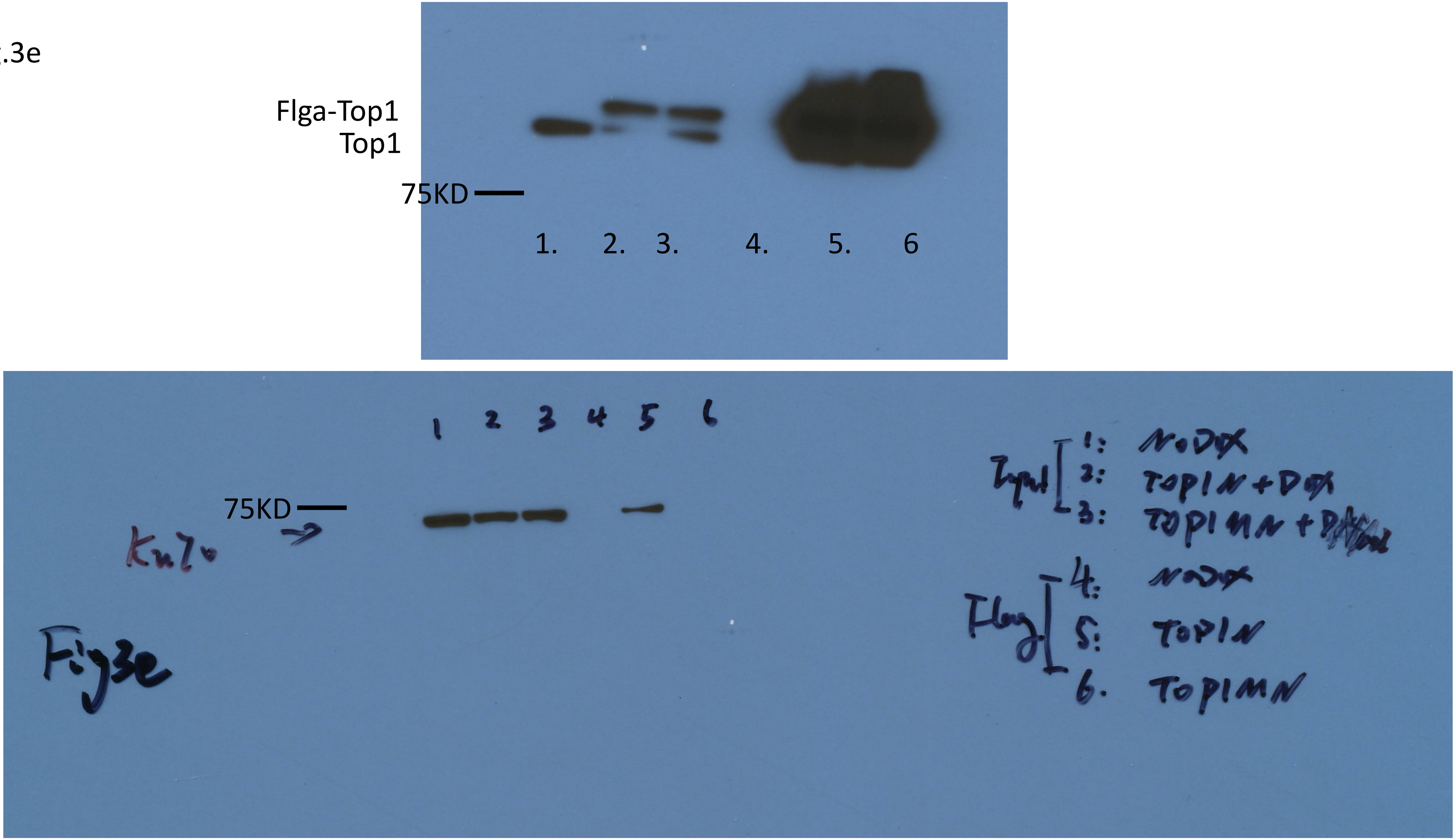

Flga-Top1
Top1
75KD
1. 2. 3. 4. 5. 6
1 2 3 4 5 6
75KD
Ku70
Fig3e
Input
1: NoDox
2: TOP1N+Dox
3: TOP1MN+Dox
Flag
4: NoDox
5: TOP1N
6. TOP1MN

Fig.3f

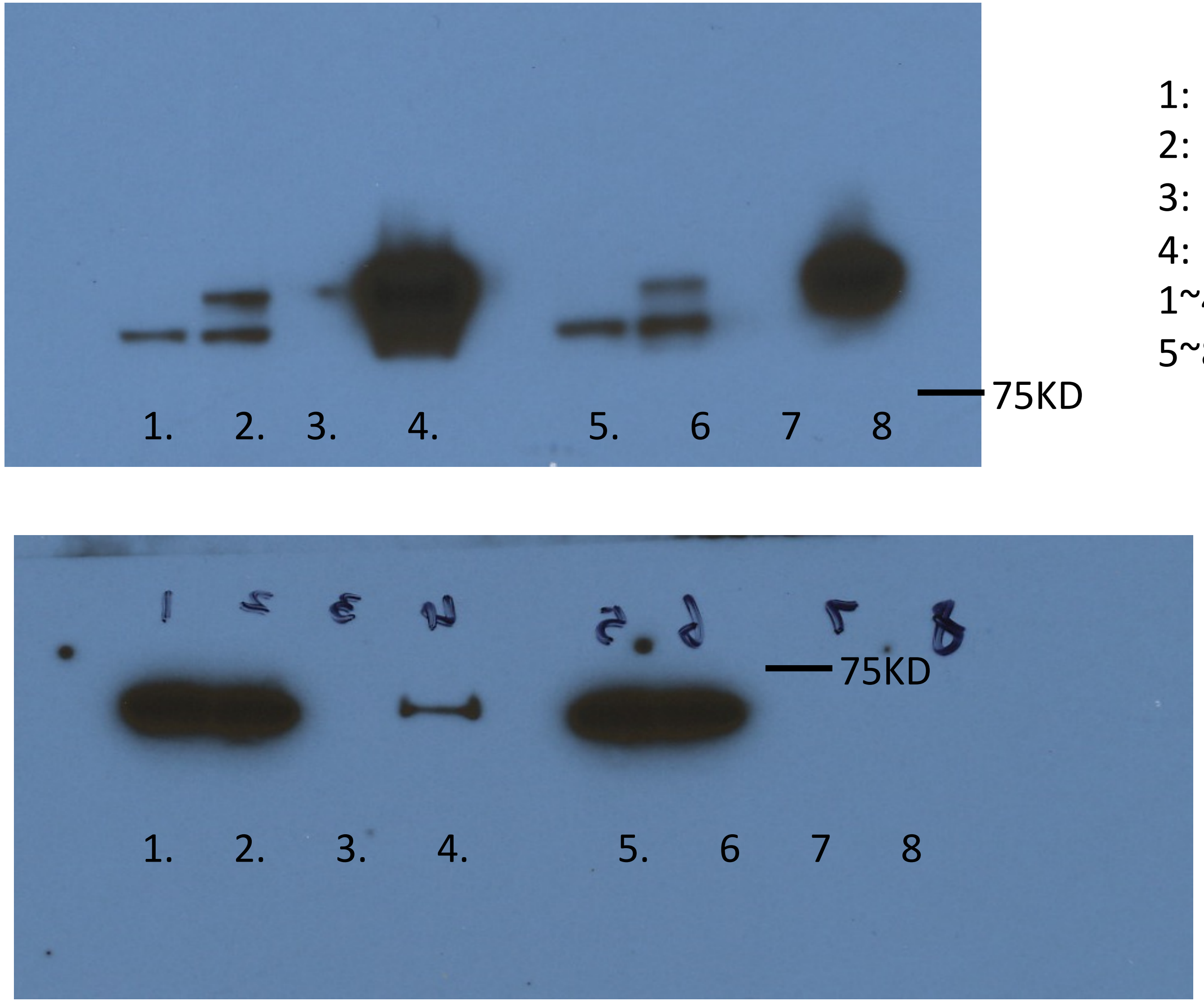

1: Input DOX-
2: Inpu DOX+
3: Flag IP DOX-
4: Flag IP DOX+
1~4: DNase-
5~8: DNase+
