# Supplementary material for: Signal-induced enhancer activation requires Ku70 to read topoisomerase1–DNA covalent complexes
Source: Nat Struct Mol Biol. 2023 Feb 6;30(2):148–58. doi: 10.1038/s41594-022-00883-8 (PMC9935399; doi:10.1038/s41594-022-00883-8)
Supplement: Source Data Fig. 3h — Source data for Fig. 3h. [file 41594_2022_883_MOESM7_ESM.pdf]

Fig.3h

Tff1e	Veh			E2		
shScr	31.8143	31.4967	31.2519	27.9261	28.1227	28.1221
shKu70	32.2257	31.1214	31.4835	30.0624	30.6331	30.5235
shXRCC4	32.5831	32.5338	33.1285	29.5653	29.2727	29.5708
shDNA-PKcs	33.052	32.6681	32.3361	31.1024	31.6195	30.5451
Greb1e	Veh			E2		
shScr	27.4643	26.8323	27.1907	24.0401	23.8902	23.751
shKu70	26.9044	26.4366	26.7304	25.5864	25.6277	25.5593
shXRCC4	27.5263	26.8499	28.0674	25.3453	25.2316	25.3664
shDNA-PKcs	28.4047	27.7524	27.5365	27.7412	26.2426	26.0034
GAPDH	Veh			E2		
shScr	19.7345	19.0887	19.5347	18.4944	18.3485	18.5649
shKu70	19.213	19.0036	19.4223	18.7529	18.9159	18.8111
shXRCC4	19.6739	19.0731	20.0043	19.7873	19.6998	19.9339
shDNA-PKcs	20.5213	20.1374	19.8392	18.5528	18.4209	18.4963
