# Supplementary figures and images for: Signal-induced enhancer activation requires Ku70 to read topoisomerase1–DNA covalent complexes
Source: Nat Struct Mol Biol. 2023 Feb 6;30(2):148–58. doi: 10.1038/s41594-022-00883-8 (PMC9935399; doi:10.1038/s41594-022-00883-8)

Fig.4c

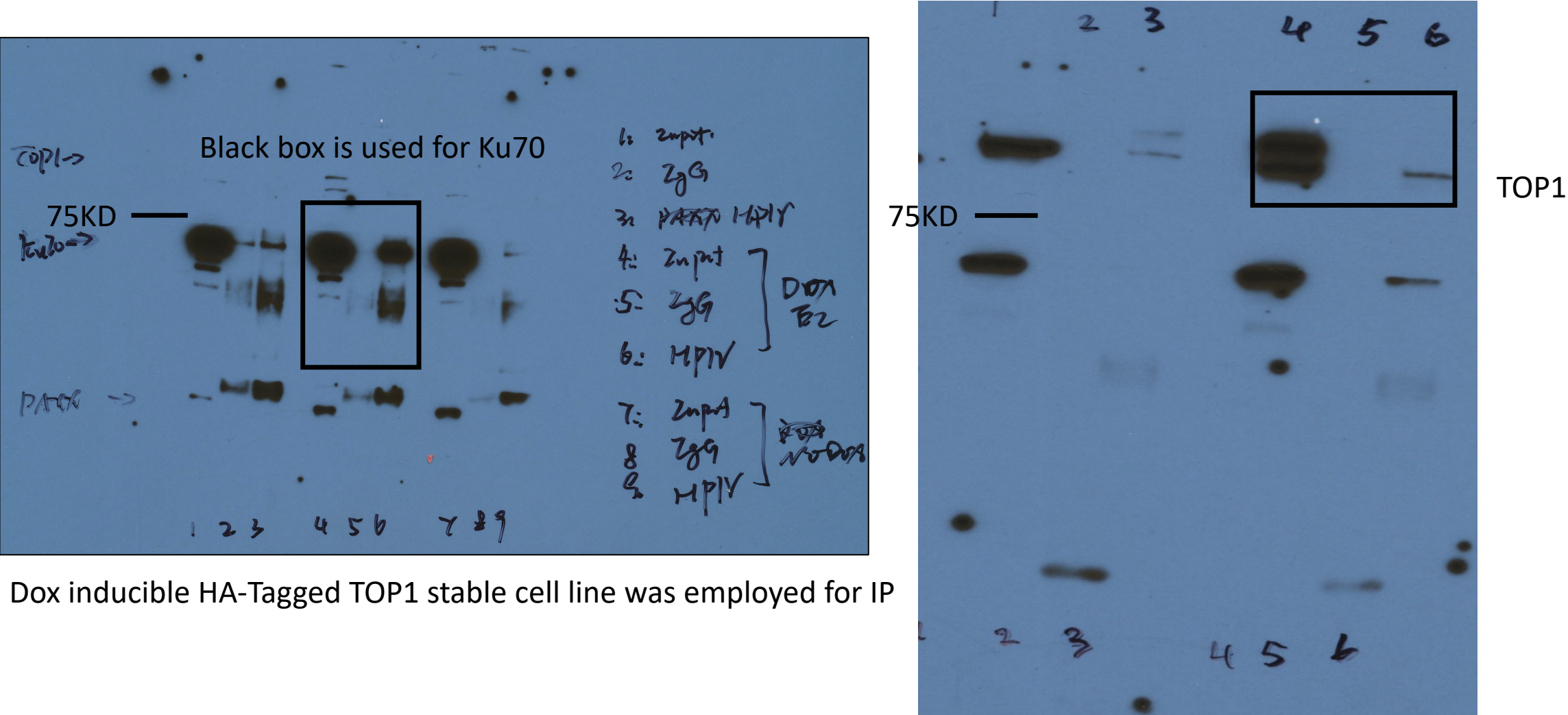

Dox inducible HA-Tagged TOP1 stable cell line was employed for IP

Supplement: Source Data Fig. 4c — Unprocessed western blots for Fig. 4c. [file 41594_2022_883_MOESM8_ESM.pdf]

Extended Data Fig.3a

siTopo1\_5UTR was not employed in this paper.

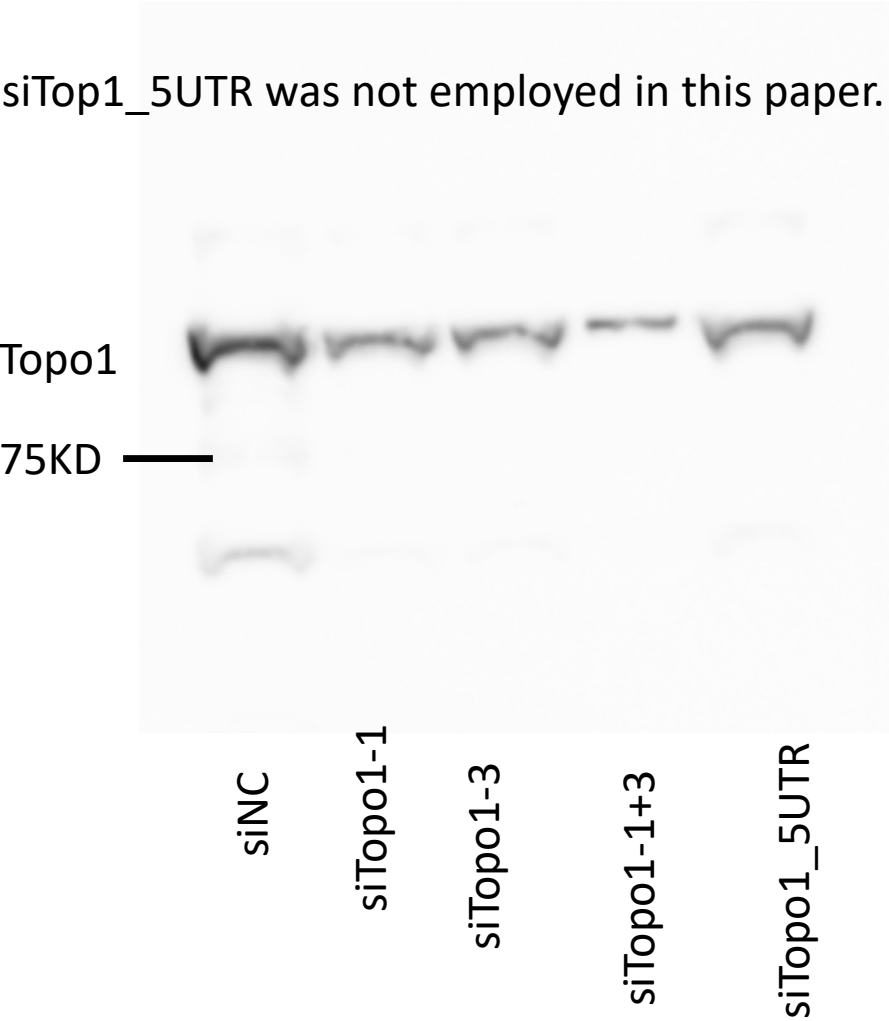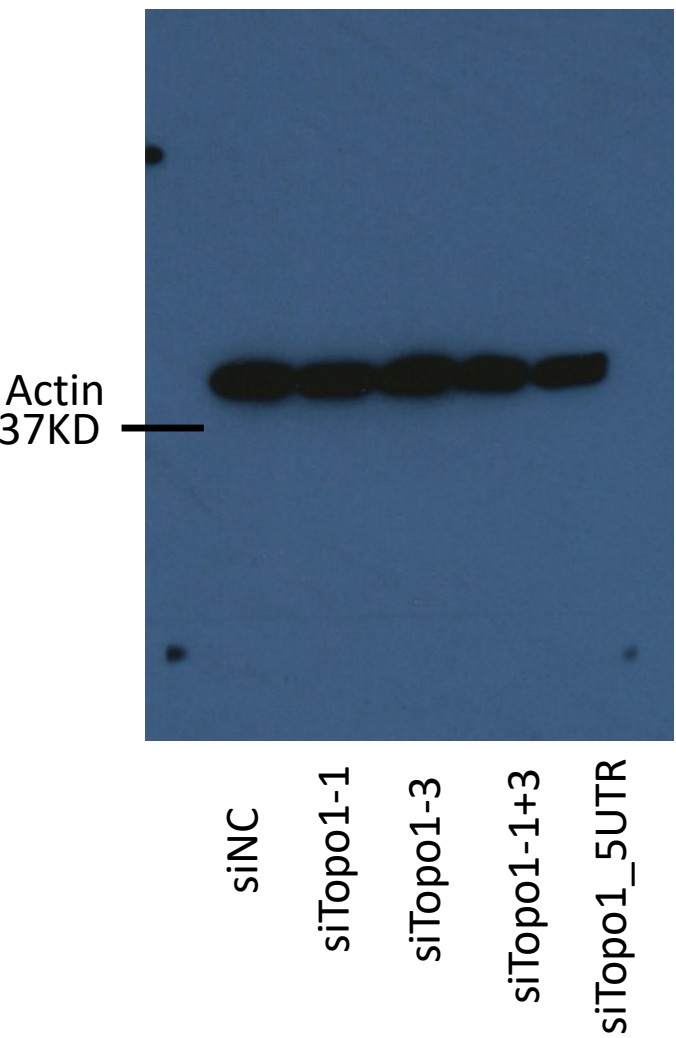

Supplement: Source Data Extended Data Fig. 3a — Unprocessed western blots for Extended Data Fig. 3a. [file 41594_2022_883_MOESM10_ESM.pdf]

Extended Data Fig.7a

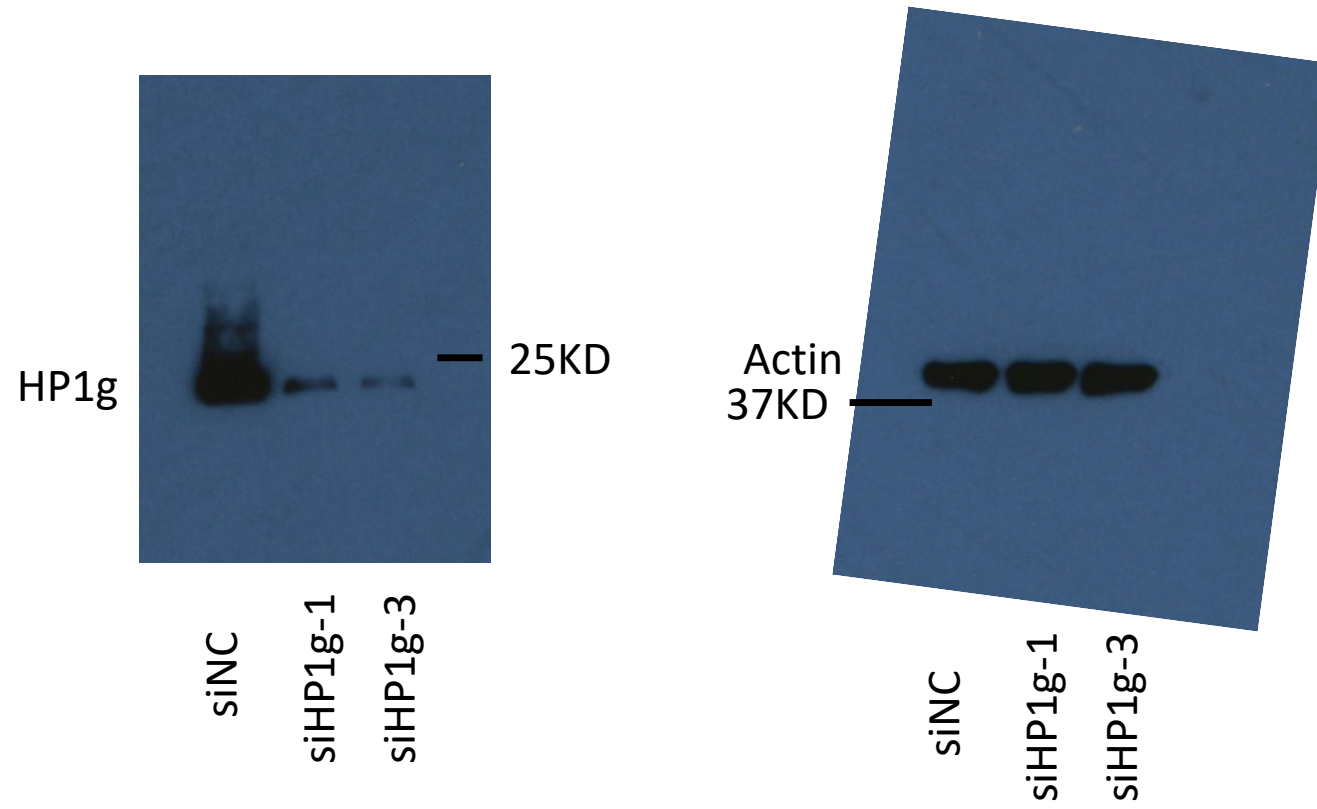

Supplement: Source Data Extended Data Fig. 7a — Unprocessed western blots for Extended Data Fig. 7a. [file 41594_2022_883_MOESM12_ESM.pdf]

Extended Data Fig.9a

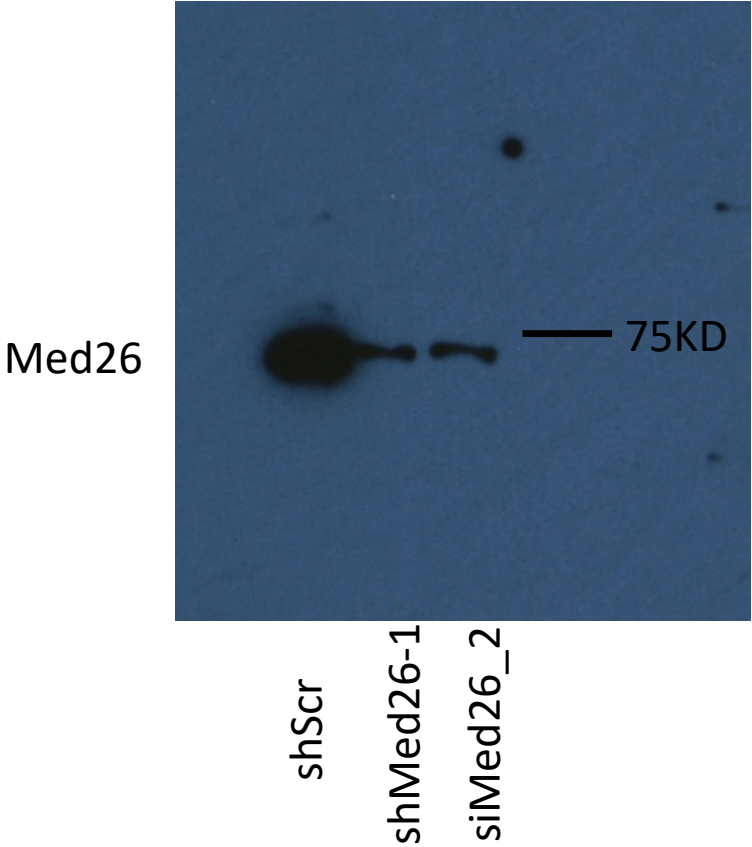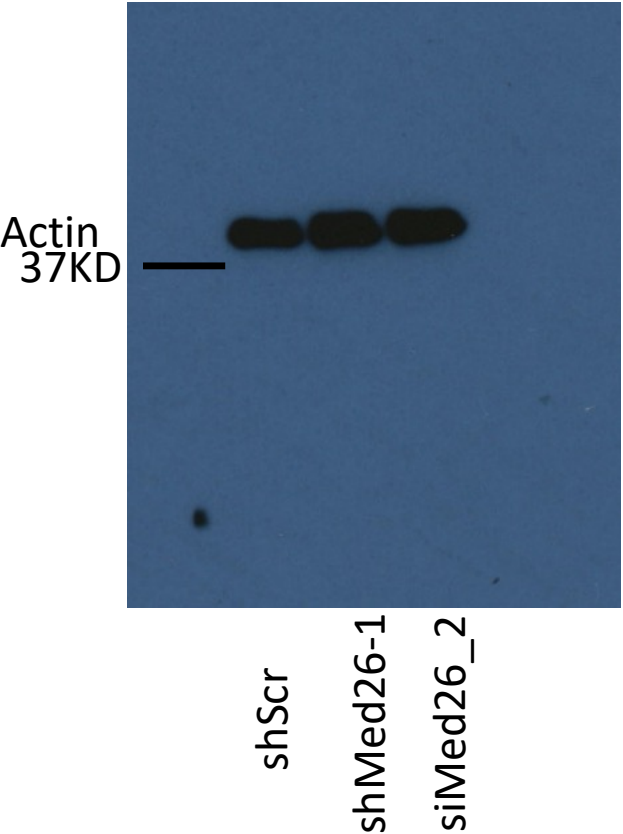

Supplement: Source Data Extended Data Fig. 9a — Unprocessed western blots for Extended Data Fig. 9a. [file 41594_2022_883_MOESM13_ESM.pdf]
