# Supplementary material for: Signal-induced enhancer activation requires Ku70 to read topoisomerase1–DNA covalent complexes
Source: Nat Struct Mol Biol. 2023 Feb 6;30(2):148–58. doi: 10.1038/s41594-022-00883-8 (PMC9935399; doi:10.1038/s41594-022-00883-8)
Supplement: Source Data Fig. 5c — Source data for LNCAP and MCF7 cells in Fig. 5c. [file 41594_2022_883_MOESM9_ESM.pdf]

Fig.5c--LNCAP

Klk2	Veh			DHT		
siNC	30.8429	30.0779	30.8475	26.9292	26.7743	27.7502
siTop1	30.7437	29.9154	29.4502	30.4851	30.6382	31.6751
siKu70	31.2806	29.4497	30.3386	31.3249	30.681	30.5935
siHP1g	31.2642	31.7862	31.1467	31.9324	30.9029	31.0302
Klk3	Veh			DHT		
siNC	23.605	22.7684	23.4504	21.1788	20.5675	22.718
siTop1	22.4749	23.3937	23.6831	23.8771	23.3746	23.8004
siKu70	23.1014	22.0506	22.8434	23.6601	23.9575	23.7042
siHP1g	22.5713	22.3234	22.7403	24.4293	23.8372	24.4099
Tmprss2	Veh			DHT		
siNC	28.404	27.6966	27.4347	23.5563	23.3138	24.9179
siTop1	27.0032	26.8498	27.4028	27.19	26.166	26.4029
siKu70	27.4875	26.5725	27.1867	26.4167	26.8637	26.5071
siHP1g	27.3213	26.9279	27.0986	26.2638	26.4952	26.4999
GAPDH-LNCAP	Veh			DHT		
siNC	19.8196	18.9167	19.3953	18.9494	18.6388	19.9525
siTop1	19.0561	18.8619	19.3036	18.6527	18.4995	18.9603
siKu70	19.5333	18.7427	19.3789	18.6662	18.8224	18.7280
siHP1g	18.8148	18.6489	18.9697	18.6467	18.7492	18.8891

Fig.5c—MCF7

IL6	Veh			TNFa		
siNC	34.4491	33.1027	33.9605	31.2763	31.1291	32.5493
siTop1	33.8697	33.9085	34.5056	32.5262	32.6892	33.6184
siKu70	34.6893	33.0385	33.3569	33.0653	33.0635	32.9509
siHP1g	35.163	35.8444	35.0157	33.6732	32.3675	32.7092
IL8	Veh			TNFa		
siNC	32.5569	32.05	32.822	23.3704	23.2838	24.8641
siTop1	32.0758	32.7941	33.4971	26.735	26.3687	26.5424
siKu70	33.114	32.0081	33.0277	26.2184	26.2161	26.1375
siHP1g	32.7965	33.593	34.0722	25.4579	25.0412	25.8382
CCl2	Veh			TNFa		
siNC	32.3363	34.8167	32.7432	21.4828	21.2678	23.0206
siTop1	31.851	31.8239	32.936	26.221	25.2572	25.2306
siKu70	32.169	31.4085	32.7722	24.857	25.366	25.0335
siHP1g	33.303	32.6807	32.4246	26.4084	26.4173	26.5838
GAPDH-MCF7	Veh			TNFa		
siNC	20.0767	20.1183	20.5495	19.9805	19.4955	20.9685
siTop1	20.1872	19.9482	20.7900	19.6630	19.5364	19.7924
siKu70	20.7744	19.8908	20.6520	19.6975	19.9253	19.7205
siHP1g	20.1602	20.0612	20.2494	19.6760	19.6165	19.8890
