# Supplementary material for: Signal-induced enhancer activation requires Ku70 to read topoisomerase1–DNA covalent complexes
Source: Nat Struct Mol Biol. 2023 Feb 6;30(2):148–58. doi: 10.1038/s41594-022-00883-8 (PMC9935399; doi:10.1038/s41594-022-00883-8)
Supplement: Source Data Extended Data Fig. 5 — Source data for Extended Data Fig. 5. [file 41594_2022_883_MOESM11_ESM.pdf]

Extended Data Fig.5

DNA-PKcs	shTRC			shDNA-PKcs		
Veh	26.242	25.8439	25.8362	30.6601	30.011	30.2162
E2	24.7328	24.7099	24.685	28.2436	28.3287	27.9441
Ku70	shTRC			shKu70		
Veh	25.4949	24.8576	25.1883	28.2596	28.0842	28.1383
E2	24.2142	23.9994	23.9469	27.7649	27.8376	27.2528
XRCC4	shTRC			shXRCC4		
Veh	26.5881	26.1151	26.2192	30.2567	30.0858	30.3248
E2	26.1592	25.8528	26.1737	30.6288	30.374	30.5167
GAPDH	shTRC			shDNA-PKcs		
Veh	19.7345	19.0887	19.5347	20.5213	20.1374	19.8392
E2	18.4944	18.3485	18.5649	18.5528	18.4209	18.4963
GAPDH	shTRC			shKu70		
Veh	19.7345	19.0887	19.5347	19.213	19.0036	19.4223
E2	18.4944	18.3485	18.5649	18.7529	18.9159	18.8111
GAPDH	shTRC			shXRCC4		
Veh	19.7345	19.0887	19.5347	19.6739	19.0731	20.0043
E2	18.4944	18.3485	18.5649	19.7873	19.6998	19.9339
